# Editorial: Sex differences in immunometabolism, prophylaxis and therapy

**DOI:** 10.3389/fimmu.2023.1259382

**Published:** 2023-08-07

**Authors:** Ashley Fink, Suresh Mishra, Milica Vujičić

**Affiliations:** ^1^ Department of Biology, College of Saint Benedict and Saint John’s University, Collegeville, MN, United States; ^2^ Department of Internal Medicine, College of Medicine, Rady Faculty of Health Sciences, University of Manitoba, Winnipeg, MB, Canada; ^3^ Department of Physiology, Institute of Neuroscience and Physiology, The Sahlgrenska Academy at University of Gothenburg, Göteborg, Sweden

**Keywords:** sex difference, metabolism, immunity, therapy, immunometabolic dysregulation

The immune system and metabolism are profoundly intertwined on a whole-body and cellular level, thus affecting homeostatic and pathological processes. Sex differences in immunometabolism have long been appreciated, yet not fully explored. The aim of this Research Topic was to reduce the knowledge gap in our understanding of sex differences in immunometabolism. Premenopausal females typically mount stronger innate and adaptive immune responses than males, which could lead to better protection against infections but also increase the risk for autoimmune disorders. As reviewed by Nytrova and Dolezal, sex bias in multiple sclerosis and neuromyelitis optica spectrum disorders affects the incidence, disease progression and risk of relapse. Biological sex also greatly affects whole-body metabolism. Premenopausal females expand subcutaneous fat whereas males, and females with polycystic ovary syndrome (PCOS), display visceral adiposity which is linked with a higher risk for metabolic disorders. Visceral adiposity is associated with chronic inflammation that attenuates immune responses to infection. It is therefore not surprising that sex differences in immunometabolism can shape responses to vaccines and therapeutic drugs. Several studies have reported that males have a higher risk for severe COVID-19 than females. As summarised in a review article by Rehman et al., this could be due to sex differences in estrogen receptor expression which can affect the HDL to LDL ratio, the nitric oxide synthesis, and others differently in males and females. Females with PCOS are also at higher risk for COVID-19 complications than non-PCOS females, further emphasising the intimate interactions between the immune system and metabolism on the outcome of infection. In a related study, Parker et al. found that induction of SARS-CoV2 antibody responses are more rapid in females. Earlier induction of an antibody response could confer faster clearance of the virus from the respiratory tract, thus explaining the sex-bias in disease severity. Sex-bias could also affect the treatment outcome. For instance, a study by Harnett et al. explored the impact of ES-62, a parasitic worm product, in a mouse model of obesity-accelerated aging. The authors found that ES-62 acts protectively in the male mice, but not in the female mice fed a high-fat diet. Specifically, ES-62 improved aging-induced loss of bone structure. This was found to be primarily through the reduction of age-associated adipogenesis in bone marrow, thus increasing osteoblast differentiation. Furthermore, ES-62 increased the level of IL-10 producing regulatory B-cells in spleen and mesenteric lymph nodes of obese male, but not female mice. The study from Grabowski et al. brings important insight into sex-specific differences in behavioural and immunological responses to antibiotic and bacteriophage administration in mice. Of note, bacteriophage administration in tested concentrations seem to be safe for both male and female mice. Contrary to bacteriophages, Grabowski et al. showed that two common antibiotics- enrofloxacin and tetracycline, significantly impaired immune responses and central nervous system activity in female mice only. Since this effect was seen very early (two weeks of antibiotic administration), authors discuss the potential effect of early antibiotic exposure on metabolic disorders. The host microbiota and their metabolites act as signals regulating systemic and local immune responses. Meng et al. studied gonadal bacterial composition in swamp eels and found a significant difference between testes and ovaries. Even though major phyla were the same between sexes, their relative abundance showed difference with e.g., *Firmicutes* being more abundant in testes than ovaries. Authors further analysed function prediction of microbial genes expressed in testes and ovaries. Whereas metabolism of amino acids, vitamins and cofactors were significantly enriched in ovaries, testes microbiota genes were enriched for carbohydrate metabolism and immune system activation. It would be of value to translate these findings to the PCOS model. In a study by Balan et al., the authors tested the effect of pregnane neurosteroid interactions with toll like receptors. While neurosteroids are present in both males and females, they are specifically elevated during luteal phase of menstrual cycle and during pregnancy, possibly preventing overt immune system activation. Balan et al. reported that allopregnanolone inhibits TLR4 signalling in human marrow derived macrophages. However, allopregnanolone inhibited TLR7 specifically in female derived macrophages. Together, this highlights the importance of including both sexes in biological research of any kind to facilitate differential therapeutic options and treatment development more precisely. On the cellular level, immune cell phenotype is intimately connected with metabolic status. In states of high energy demand immune cells rely on glycolysis for producing ATP, while oxidative phosphorylation is the preferred energy source in resting and regulatory states. Along this line, Alharshawi et al. found that monocyte infiltration upon alcohol induced liver injury is dependent on interferon alpha receptor in females only. This study provides a cellular mechanism that could explain the sex disparity in alcohol induced liver injury that is more typical for women. On a similar note, Scott et al. explored the gain of function mutation in STAT1 by generating a mouse model carrying human STAT1 with the T385M mutation. Humans with the T385M mutation in STAT1 are more prone to autoimmunity, yet the mechanisms are not fully understood. Authors show that Stat1^t385m/+^mice, in the absence of infection, display aberrant an adaptive immune response, with disrupted homeostasis and enhanced activation of T helper lymphocytes and atypical B cell activation, collectively leading to autoimmunity. Interestingly, these processes occurred earlier and were more robust in females. It would be interesting to assess cellular metabolism of such T helper and B lymphocytes, since it is known that STAT1, besides interferon signal transmission, regulates glycolysis, TCA cycle and oxidative phosphorylation. Crosstalk of interferon pathways and mitochondria is linked through mitochondrial NOD-like receptor X1 (NLRX1), as demonstrated in the study by Snäkä et al. Here the authors used the model of infection with parasitic worm *Leishmania guyanensis*, and showed that NLRX1 attenuated inflammation in females but not in males. Nlrx1-deficient macrophages from females were skewed towards a masculine phenotype with higher rates of glycolysis and OXPHOS, coupled with increased type I interferon production

In conclusion, we believe that this Research Topic expands our knowledge of sex-difference in immunometabolism. Understanding how biological sex can shape immune and metabolic responses will facilitate development of novel targets in immunometabolic disorders, targeted lifestyle interventions and modulations of existing therapies.

## Author contributions

AF: Writing – original draft, Writing – review & editing. SM: Writing – original draft, Writing – review & editing. MV: Writing – original draft, Writing – review & editing.

